# Unraveling the complex relationship between prenatal alcohol exposure, hippocampal LTP, and learning and memory

**DOI:** 10.3389/fnmol.2023.1326089

**Published:** 2024-01-12

**Authors:** Monica Goncalves-Garcia, Derek Alexander Hamilton

**Affiliations:** Department of Psychology, University of New Mexico, Albuquerque, NM, United States

**Keywords:** neurodevelopment, neuroplasticity, glutamate, LTP, cognition, memory, hippocampus

## Abstract

Prenatal alcohol exposure (PAE) has been extensively studied for its profound impact on neurodevelopment, synaptic plasticity, and cognitive outcomes. While PAE, particularly at moderate levels, has long-lasting cognitive implications for the exposed individuals, there remains a substantial gap in our understanding of the precise mechanisms underlying these deficits. This review provides a framework for comprehending the neurobiological basis of learning and memory processes that are negatively impacted by PAE. Sex differences, diverse PAE protocols, and the timing of exposure are explored as potential variables influencing the diverse outcomes of PAE on long-term potentiation (LTP). Additionally, potential interventions, both pharmacological and non-pharmacological, are reviewed, offering promising avenues for mitigating the detrimental effects of PAE on cognitive processes. While significant progress has been made, further research is required to enhance our understanding of how prenatal alcohol exposure affects neural plasticity and cognitive functions and to develop effective therapeutic interventions for those impacted. Ultimately, this work aims to advance the comprehension of the consequences of PAE on the brain and cognitive functions.

## Introduction

Deficits following prenatal alcohol exposure (PAE) have been extensively investigated for decades. It is widely accepted that binge drinking results in a series of deficits, including morphological and cognitive alterations. However, less is known about impairments associated with moderate PAE. Over the years, significant progress has been made in understanding the neurobiological mechanisms underlying and contributing to the expressions of behaviors that are impaired following PAE. Given the similarities across the mammalian brain (Gil-Mohapel et al., 2010; Patten et al., 2014), rat models of PAE are essential and valid for elucidating the effects of PAE on the mechanisms involved in the brain plasticity (Fontaine et al., 2016). Furthermore, this research may contribute valuable insights for the development of interventions for the clinical population.

The overarching goal of this review is to offer a comprehensive exploration of the current research on the impacts of PAE, with a particular focus on moderate levels of exposure when data is available. This review aims to delve into the neurobiological basis of learning and memory processes affected by PAE. The objective is to establish a coherent understanding across different domains. It is evident that even low levels of prenatal alcohol exposure can lead to enduring effects on exposed individuals. While it is widely recognized that the hippocampal formation is particularly sensitive to developmental alcohol exposure, there are significant gaps remaining in understanding impairments of neurobiological mechanisms and subsequent cognitive manifestations. This review seeks to provide a foundational framework for further investigation, with the goal of comprehending the underlying mechanisms behind the negative impacts of PAE on learning and memory, enabling early diagnosis and therapeutic interventions for affected individuals.

## Neural bases of learning and memory

The ability of the brain to adapt to experience and the mechanisms involved in the strengthening - or weakening–of neural connections has been investigated for over 100 years (Bliss and Collingridge, 1993; Nicoll, 2017). Synaptic plasticity can be defined as an activity-dependent alteration of the strength of synapses. The long-lasting forms of synaptic plasticity include long-term potentiation (LTP) – persistent strengthening of synapses - and long-term depression (LTD, *for review see* Fontaine et al., 2016) – persisting decreasing in the strength of synapses. In the early 70s, there was a breakthrough in understanding the neural mechanisms underlying synaptic plasticity with the discovery of LTP in the granule cells of the dentate gyrus following high-frequency stimulation (HFS) to the rabbit perforant pathway (Bliss and Lømo, 1973). Following the publication of that study, significant progress has been made in the advancement of the understanding of the cellular and molecular mechanisms underlying synaptic plasticity. The phenomenon of LTP has been identified at synapses across the brain (Lynch, 2004), however, it is mostly studied in the hippocampus as the area plays a major role in spatial navigation, learning, and memory (Bonthius et al., 2001). Investigations of LTP have been extensively conducted at the hippocampal formation as the discoveries associated with LTP provide evidence for the cellular basis of learning and memory (Larson and Lynch, 1986; McNaughton et al., 1986; Bashir et al., 1993; Sutherland et al., 1997; Lynch, 2004; Nicoll, 2017).

### Properties of LTP

Long-term potentiation requires simultaneous depolarization of the pre-and post-synaptic terminals. The basic properties of LTP are cooperativity (co-activation of multiple excitatory synapses simultaneously or in close temporal proximity), associativity (weak stimulation of a single pathway can induce LTP if strong stimulation of another pathway is delivered simultaneously), and input specificity (occurrence of LTP at a single synapse without spreading to others). Together these properties ensure the accuracy of memory storage and maintenance (Abraham et al., 2019).

**FIGURE 1:**
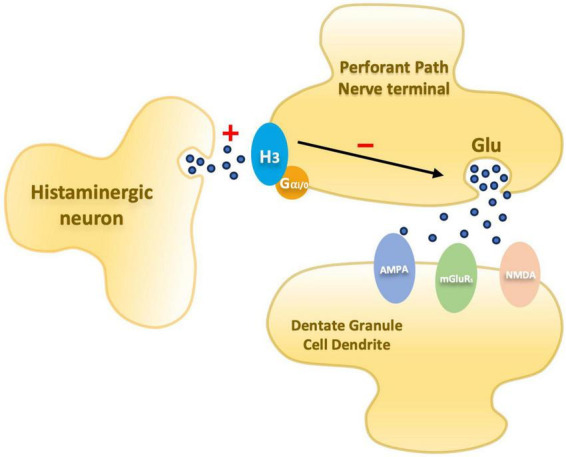
Schematic diagram illustrating an entorhinal perforant path nerve terminal and postsynaptic dentate granule cell dendrite. Presynaptic histamine H_3_ receptors mediate inhibition of glutamate release. In PAE animals the inhibitory mechanism is elevated.

Briefly, the mechanisms underlying LTP are mediated by the release of the excitatory neurotransmitter glutamate – which plays a crucial role in the mammalian brain by facilitating most excitatory transmission and by its involvement in cognition, learning, and memory – and the activation of the glutamate receptors NMDA (Collingridge et al., 1983) and AMPA in the postsynaptic cell (Bordi, 1996; Bliss et al., 2018). During low-frequency stimulation, glutamate from the presynaptic terminal binds to NMDAR and AMPAR channels in the postsynaptic terminal (Figure 1). The AMPAR channel is open and allows for the flow of Na + and K + . The NMDAR channel is blocked by Mg2 + which is removed following HFS allowing, then, for Ca^2+^ to flow through the channel (Nicoll, 2017). However, NMDAR activation is not necessary for the induction of all forms of LTP (Bashir et al., 1993; Lynch, 2004) in the CA1 region. Unlike NMDA receptor-dependent LTP, mossy fiber LTP can occur independently of NMDA receptors. Notably, inducing LTP in CA1 without NMDA receptors is possible, but generally, Schaffer-commissural LTP requires NMDAR (Lynch, 2004). Ongoing research explores mossy fiber LTP’s mechanisms, enduring changes, and diverse roles across brain regions (Malenka and Bear, 2004; Nicoll and Schmitz, 2005).

In addition, other features of synaptic transmission are NMDA-receptor independent, such as paired-pulse facilitation (PPF) - an enhanced response to two closely spaced stimuli; provides insights into short-term synaptic plasticity and the interplay between presynaptic and postsynaptic mechanisms in neurotransmission studies - and post-tetanic potentiation (PTP) - a short-term enhancement of synaptic transmission observed after a brief period of high-frequency stimulation, reflecting the heightened release of neurotransmitters at the synapse (Bliss and Collingridge, 1993). To investigate the involvement of the neurotransmitter glutamate in the induction of LTP, studies investigated hippocampal LTP in mice lacking mGlu1 – a glutamate receptor subtype. The results suggest a reduction in LTP compared with control animals which supports the implication of glutamate receptors in the LTP induction (Bashir et al., 1993; Bordi, 1996). NMDAR is referred to as a “coincidence detector” because of the necessity to activate both presynaptic glutamate release and postsynaptic depolarization (Citri and Malenka, 2008). LTP is comprised of early (e-LTP) and late (l-LTP) phases. The former is independent of protein synthesis and lasts for about 1 h, while the latter requires *de novo* protein synthesis for the maintenance of the LTP. Briefly, e-LTP is frequently understood as being the outcome of a single HFS episode and of shorter duration (∼1 h). During the induction phase, increased synaptic input triggers the activation of various signaling pathways. Calcium influx into the postsynaptic neuron activates Ca2 + /calmodulin-dependent protein kinase II (CaMKII) and other calcium-dependent kinases. These kinases, in turn, phosphorylate AMPA receptors, facilitating their trafficking to the postsynaptic membrane. Additionally, protein kinase C (PKC) is activated, contributing to the modulation of synaptic efficacy. These phosphorylation events play a crucial role in the expression of LTP, where the strengthened synaptic connections are maintained (Citri and Malenka, 2008; Fontaine et al., 2016; Herring and Nicoll, 2016; Tao et al., 2021). The early phase sets the stage for the subsequent molecular and structural changes that underlie the long-lasting modifications associated with LTP, ultimately contributing to the cellular basis of learning and memory. l-LTP requires multiple episodes of HFS and is dependent upon the activation of protein kinase A (PKA) and CaMKII, which develops over time and could last for several hours. Gene transcription and protein synthesis (Malenka and Bear, 2004) contribute to the synthesis of new proteins necessary for maintaining the strengthening of the synapses. PKMzeta has been linked to memory (Pastalkova et al., 2006) and maintenance of LTP through trafficking and expression of AMPARs (Yao et al., 2008; Bingor et al., 2020) and NMDARs (Shema et al., 2007; Yao et al., 2008). Other potential processes that contribute to induction and maintenance of LTP include retrograde signaling of Nitric Oxide (NO) (Bon et al., 1992; Mizutani et al., 1993), extra synaptic AMPARs and spillover of glutamate (Kullmann and Asztely, 1998), and modified sensitivity of metabotropic glutamate receptors (Aronica et al., 1991). The reader is referred to the reviews of Herring and Nicoll (2016) and Hayashi (2022) for broader treatment of LTP mechanisms.

Late-long-term potentiation requires multiple episodes of HFS and is dependent upon the activation of protein kinase A (PKA) and CaMKII, which develops over time and could last for several hours. Gene transcription and protein synthesis (Malenka and Bear, 2004) contribute to the synthesis of new proteins necessary for maintaining the strengthening of the synapses. Some studies of *in vivo* LTP showed evidence for the induction of LTP that lasted for several hours following a single instance of HFS (McNaughton et al., 1986; also see Park et al., 2014 for additional details on different forms of LTP that are dependent on the timing of the stimuli and are pharmacologically distinct).

Given that multiple actions of distinct nature and location must take place for potentiation to occur, identification of those poses a challenge when attempting to understand possible alterations following developmental insults. The first conclusions about LTP were that it was purely a postsynaptic mechanism. However, with the emergence of more research in the matter of nature and loci of expression, the conclusions gravitated between pre- or post-, to a mix of both, to both pre-and postsynaptic mechanisms (Bliss and Collingridge, 1993) that characterize different types of LTP. Presynaptic mechanisms like H3 receptors and mGluRs (metabotropic glutamate receptors) participate in regulating neurotransmitter release and represent promising targets for manipulation and interventions aimed at modulating long-term potentiation. Additionally, PAE can lead to different long-lasting alterations in synaptic plasticity mechanisms that includes reduced neurogenesis and cell loss (Choi et al., 2005; Gil-Mohapel et al., 2010; Yang et al., 2017; Bird et al., 2018), neuronal morphology and spine architecture (Berman and Hannigan, 2000; Hamilton et al., 2010; Mira et al., 2020), and NMDAR level alterations (Hughes et al., 1998; Samudio-Ruiz et al., 2010; Bird et al., 2020; Plaza-Briceño et al., 2020). These are some of the mechanisms that could be altered by PAE and have a negative impact on synaptic plasticity. Understanding the deficits and mechanisms interactions are key to develop potential interventions that could mitigate those long-lasting impairments.

### LTP and prenatal alcohol exposure

Animal models of PAE using *in vitro* or *in vivo* electrophysiology to investigate LTP in the hippocampal formation suggest that there are fundamental alterations in the neural mechanisms when compared to non-exposed animals. Even though lower levels of PAE do not result in physical deficiencies, studies on the neurobiology of learning and memory processes have identified cognitive impairments following moderate PAE (Sutherland et al., 1997). Significant decreases in LTP have been observed in the dentate granule cells following high-frequency stimulation [HFS – trains, or tetanus, of stimulation, or theta burst stimulation (TBS)] in the perforant pathway (Sutherland et al., 1997; Varaschin et al., 2010; Brady et al., 2013; Harvey et al., 2020). While CA1 LTP has been extensively studied, the dentate gyrus (DG) is of particular interest as the primary region within the hippocampal formation to receive input from the entorhinal cortex via the Perforant pathway (PP). Furthermore, studies on DG LTP have consistently reported alterations following prenatal alcohol exposure (Sutherland et al., 1997; Varaschin et al., 2010; Titterness and Christie, 2012; Brady et al., 2013).

One of the first studies reporting electrophysiological impairment following PAE used paired-pulse facilitation in hippocampal slices, specifically in the CA1 region, with interpulse intervals varying from 5 to 400 μs (Hablitz, 1986). The results showed diminished paired-pulse response inhibition at shorter inter-pulse intervals (5–100 μs) – which involves activation of recurrent inhibitory pathways – in the PAE group compared to controls, but similar potentiation at longer intervals (200–400 μs) (Hablitz, 1986). Similar results were reported in a replication study expanding to two levels of ethanol exposure. One group of animals received a high dose of ethanol [35% ethanol-derived calories (EDC)], while the other received a low dose (17.5% EDC) group. The results were similar to the first study but only in the 35% EDC group at lower PP intervals. The 35% EDC group also showed little evidence of LTP compared to the other diet conditions (Tan et al., 1990). In contrast, another study, using a different method of alcohol administration (intragastric gavage – GD8-21–0, 4, or 6 g/kg/day) and brain slices from two age groups (PN25-32 and PN63-77), reported no difference in input/output profiles or paired-pulse responses at any group. There was a significant reduction in the amplitude of the maximal evoked population spike (PS) in the higher dose in the younger group compared to the other two groups (Krahl et al., 1999; Berman and Hannigan, 2000). This result is similar to the results reported by Sutherland et al. (1997) using a 5% ethanol liquid diet, investigating *in vivo* DG LTP in 5-month-old rats. In terms of timing, a study showed that the effects of developmental alcohol exposure yield different outcomes in DG LTP. PAE during GD10-21 (2nd trimester-equivalent) only resulted in less potentiation while exposure during GD1-9 and PN1-9 (1st and 3rd trimester-equivalent) did not have a significant effect (Helfer et al., 2012). While these results support timing as a factor, there is still a need for clarification on the teratogenic effects of alcohol. During the 2nd trimester equivalent, the DG granule cells and interneurons undergo neurogenesis. Exposure during the early stages of development may alter cell migration, neuron and glial proliferation, and the formation of neural networks. In addition to the timing of exposure, the pattern/route of ethanol administration, as well as age during the assessment, are important factors to consider when interpreting the results from different studies of similar investigations as those may lead to diverse outcomes (Table 1).

**TABLE 1:** LTP protocols on the DG from different studies show the variability of the alcohol exposure paradigm, stimulation protocol, and stimulation outcomes.

Alcohol exposure paradigm/BAC	Stimulation paradigm	Effects	References
**Prenatal Alcohol Exposure and Plasticity Effects on Brain Areas**			
Liquid Diet; GD 1–22; 5% v/v; BAC 83.2 mg/dL	Pulse frequency: 30 s Induction: 10 × 400 Hz	LTP M ↓	Sutherland et al., 1997
Liquid Diet; GD 1–22; 6.61% v/v; BAC 184 mg/dL	Pulse frequency: 15 s Induction: 5 × (10 × 5 Pulses, 100 Hz)	LTP M ↓	Christie et al., 2005
Liquid Diet; GD 1–22; 5% v/v; BAC 84 mg/dL	Induction: 3 × 400 Hz, 25 ms or 10 × 400 Hz, 25 ms	LTP M ↓with 3 × 400 Hz protocol	Varaschin et al., 2010
Liquid diet; GD11–21; 6.6% v/v; BAC 142 mg/dL	Pulse frequency: 30 s Induction: HFS 4 × 50 pulses, 100 Hz or TBS 5 x (4 × 4 Pulses, 100 Hz)	LTP M ↓	Helfer et al., 2012
Drinking Water; GD 1–22; 5% v/v; BAC 84 mg/dL	Induction: 4 × (10 × 5 Pulses, 400 Hz)	LTP M↓; F-	Patten et al., 2013a
Liquid Diet; GD 1–22; 6.61% v/v; BAC 101.5 mg/dL	Induction: 4 × (10 × 5 Pulses, 400 Hz)	LTP M ↓; F-	Patten et al., 2013b
Liquid Diet; GD 1–22; 6.6% v/v; BAC 146.32 mg/dL	Induction: 4 × (10 × 5 pulses, 400 Hz)	LTP M↓; F-	Sickmann et al., 2014
Drinking Water; GD 1–22; 5% v/v; BAC 84 mg/dL	Pulse Frequency: 30 s Induction: 3 × 400 Hz	LTP M ↓	Varaschin et al., 2014
Liquid diet; GD1–22; 6.6%v/v; BAC 80–180 mg/dL	Pulse frequency: 30 s Induction: 4 trains x 50 pulses, 100 Hz	LTP M ↓; F↓	Fontaine et al., 2019
Alcohol solution; GD1–PD7; 10% v/v; BAC: 62 mg/dL	Pulse frequency: 30s Induction: HFS 8 x (3 × 8 pulses, 200 Hz)	LTP M ↓	Plaza-Briceño et al., 2020
Liquid diet; GD1–22; 6.6% v/v; BAC 80–180 mg/dL	Pulse frequency: 30 s Induction: HFS 4 × 50 pulses, 100 Hz	LTP M ↓; F-	Grafe et al., 2022

This table shows the reduced number of studies on LTP on the DG following throughout the years.

### Histaminergic and glutamatergic transmissions

Over the years, since the discovery of LTP, significant progress has been made in understanding the components of the complex neurobiological network supporting synaptic plasticity. As a result, a better understanding of what may be altered by prenatal insults has emerged offering potential avenues for interventions. Research in this field suggests that there is a disruption in a presynaptic component of LTP involved in the glutamate release (Varaschin et al., 2010, 2014, 2018) mediated by H3 receptors (H_3_R). These receptors are autoreceptors – modulating histamine release – that also acts as heteroreceptors modulating release of other neurotransmitters, including glutamate. Passani et al. (2004), reviewed the role of the histamine H_3_R as a possible target for pharmacological interventions to enhance cognition and treat possible disorders associated with sleep, stress, and anxiety. The researchers highlight the possible association of the histaminergic system in cognitive processes and describe results from studies using H_3_R antagonists and inverse agonists that improved cognitive performances in cognitively impaired animals. Together, these suggest that neurotransmitters modulated by H_3_R are involved in cognition and that H_3_R antagonists or inverse agonists could potentially reverse cognitive deficits (Passani et al., 2004). In the dentate gyrus, H_3_Rs are located on the perforant pathway and have been demonstrated to inhibit glutamate release via histamine-mediated depression in calcium influx during the presynaptic action potential (Brown and Haas, 1999; Figure 1).

Glutamatergic neurotransmission is part of the presynaptic components of LTP and may be involved in activity-dependent potentiation deficits following PAE (Savage et al., 1998, 2010; Varaschin et al., 2014). Studies have investigated glutamate receptor release by utilizing a glutamate reuptake inhibitor that allowed for the measurement of the electrically evoked release of [^3^H]-D-ASP in brain slice preparations (Savage et al., 1998, 2001). Results suggest activity-dependent potentiation of D-ASP release following moderate PAE suggesting impairment in complex activity-dependent modifications in the neurotransmission (Savage et al., 1998). Even though the presynaptic neurochemical basis for this impairment remains unclear, studies established that one of the neurochemical mechanisms underlying deficits associated with PAE is a reduction in glutamate receptor-mediated potentiation of glutamate release at the synapses of the dentate granule cells (Galindo et al., 2004; Savage et al., 2010).

### Sex differences

Male animals are more commonly used in studies investigating synaptic plasticity, with fewer studies exploring both male and female animals or exclusively female animals (Titterness and Christie, 2012; Sickmann et al., 2014; An and Zhang, 2015). These studies focused on synaptic plasticity in adolescent rats (∼PND30-35) and reported bidirectional findings. PAE animals showed reduced LTP after HFS relative to control and pair-fed groups, while females exhibited enhanced LTP compared to the other groups. Titterness and Christie (2012) suggested that sex differences could be due to sex-specific alterations to NMDAR-dependent DG LTP. Subsequent work from the same group expanded on the investigation in NMDAR function and expression, but did not support sex differences. Additionally, they did not find sexually dimorphic effects on DG LTP in adulthood (PND 55–70) (Sickmann et al., 2014). Another study, including only female rats, reported enhanced LTP compared to the control groups (An and Zhang, 2015). The first two groups mentioned, used a liquid diet (35% EDC) and Sprague-Dawley rats, while the last used oral gavage in Wistar rats.

The limited number of studies investigating sexual dimorphism in LTP does not provide sufficient information to (1) accept that sex differences are significant; (2) conclude that the differences reported in adolescence, but not in adulthood, have an impact on plasticity and/or behavioral expression later in life; (3) understand the source of the differences; or (4) elucidate how sex differences may or may not be related to strain, route of ethanol administration, and – timing. Further investigations should focus on expanding on current plasticity models to include both sexes while decreasing possible confounding factors related to diet, route/timing of administration, and animal strain.

Behavioral tasks investigations show that males are more impaired than females in the probe trials of the Morris Water Task (MWT), but both are impaired in the acquisition phases of the test. While other studies report the opposite effect (Berman and Hannigan, 2000). In open-field tests, PAE male rodents are typically more hyperactive than females (Osterlund Oltmanns et al., 2022), show more perseveration errors during reversal learning in the MWT (Rodriguez et al., 2016), and exhibit spatial information retention impairment (Rodriguez et al., 2016). Studies PAE consistently show parallels between animal and human data. Hamilton et al. (2003) found that PAE children, like rodents, had greater distances in a virtual Morris Water Task. Woods et al. (2018) using a similar setup with fMRI, observed longer latencies in PAE boys but no differences in girls. Dodge et al. (2019) found deficits in place learning for syndromal boys and girls, and non-syndromal girls, but not in low-to-moderate PAE. All studies note differences in place learning, aligning with findings in animal models (Goodlett and Johnson, 1997; Dodge et al., 2020).

These differences can also be related to sexual dimorphisms in neural development. A study by Hamilton et al. (2010), shows greater PAE effects on dendritic morphology, structural plasticity, and IEG expression in males than in females. Sexual dimorphisms have been reported in hippocampal neurotransmissions and LTP (Sickmann et al., 2014; Osterlund Oltmanns et al., 2022). Although there has been numerous evidence on sexual dimorphisms and how PAE may affect males and females differently, a thorough discussion on sexual dimorphisms is beyond the intended scope of this review. It is important, however, to stress that possible sex differences must be taken into consideration as investigation on just one or the other sex may or may not reveal subtle differences between sexes.

A recent study by Stockman et al. (2022) investigated neurogenesis in the neonatal rat hippocampus and found evidence for a sexually dimorphic epigenetically based regulation of neurogenesis – specifically in the DG. Their results suggest that there is a developmental sex difference in DG cell genesis. It suggests that there is a modulatory DNA difference with elevated methylation in the males and elevated histone acetylation in females – which suppresses neurogenesis. They also state that in adulthood the overall size of male and female hippocampus does not differ. The early developmental differences – as female neurons mature earlier than male neurons – evidenced in enhanced learning and LTP in females compared to males, reverse with the achievement of reproductivity maturity (Le et al., 2022; Stockman et al., 2022). A potential explanation for sex differences in LTP and behavioral task, has been linked to possible differences in the composition of GABAa receptors before and after puberty in females. Further investigators in the field should take this information into account when interpreting the results of possible sex differences, particularly if those hippocampal-dependent tests are performed before or after adolescence. Considering the new discoveries about neurogenesis and sex differences, researchers should be mindful of the timing of exposure/assessment to have a better interpretation of results showing sex differences. Those changes could be associated with the onset of the estrous cycle. Alternatively, late maturation of interneurons and related connections, could be involved in the sex differences in hippocampal LTP before and after puberty (Le et al., 2022). Additionally, recognizing fundamental sex differences in brain development is crucial for understanding how and when these distinctions manifest. This knowledge is essential for informing the development and direction of potential interventions.

### Non-pharmacological interventions

A growing body of research focuses on potential therapies following PAE and prevention of the possible outcomes by nutraceutical interventions. Choline supplementation has been of interest because it is also involved in the formation of the neurotransmitter acetylcholine. Research has shown that choline supplementation during pregnancy has positive benefits on cognitive scores which could be a potential prenatal intervention to minimize or prevent Fetal Alcohol Spectrum Disorders (FASDs). Because individuals do not normally get diagnosed until later, there are questions about the benefits of postnatal supplementation. Animal models of PAE investigated the benefits of postnatal choline supplementation and the results showed improvement in the MWT (Grafe et al., 2022). In the pediatric clinical population, the results of postnatal supplementation have been unclear as the results seem to differ according to the age group (Wozniak et al., 2020; Grafe et al., 2022). The data on choline supplementation is still scarce. There is a general agreement that choline supplementation is possibly acting on altering hippocampal cholinergic functioning. However, choline can also influence methylation patterns and is linked with other signaling pathways, which are known to be altered by alcohol exposure (Monk et al., 2012). More investigation is needed to (1) understand the mechanisms of action of choline supplementation, (2) determine the benefits of perinatal and postnatal supplementation, (3) assess the long-lasting benefits of choline, and (4) define the ideal window of possible interventions.

Other possible interventions include - but are not restricted to - omega-3 fatty acid supplementation (Patten et al., 2013b), vitamin E, betaine, folic acid, methionine, zinc (Sebastiani et al., 2018; Maya-Enero et al., 2021), voluntary exercises (Christie et al., 2005), enriched environment (animal model) (Hamilton et al., 2014; Kajimoto et al., 2016; Gursky and Klintsova, 2017) to diminish alcohol-induced changes to the hippocampus. In addition, studies in epigenetics – alterations in gene expression that can be influenced by environmental factors – suggest that alterations in basic cellular processes following PAE may be related to long-lasting effects that include dendritic development and synaptic plasticity, as suggested by reports of reduced hippocampal cell numbers in FASD models (Varadinova and Boyadjieva, 2015; Stockman et al., 2022). These modifications in gene expression alterations include DNA methylation, histone modification, and non-coding RNA regulation, and can disrupt the development of the nervous systems leading to long-lasting impairments (Varadinova and Boyadjieva, 2015; Ehrhart et al., 2019). These may lead to disruption in the induction and maintenance of LTP, for example. More research is needed to understand the effects of alcohol on gene expression alterations. These are promising outcomes, but more research is needed to understand the possible benefits of the mechanistic bases of those effects.

### Pharmacological manipulations

Pharmacological manipulations with agents that have known receptor interactions can provide insights into receptor-level mechanisms and identify potential pharmacotherapeutic interventions. “Procognitive agents” have been shown to facilitate learning and memory (Sadek et al., 2016) and used to examine potential developmental alterations in histaminergic and glutamatergic neurotransmissions following PAE. Studies have specifically investigated the effects of the histamine H_3_R antagonist ABT-239 on both LTP and spatial navigation tasks in PAE animals (Savage et al., 2010; Varaschin et al., 2010). Assessment of the effects of the ABT-239 agent in moderate PAE animals in the MWT showed that the escape latency differences (PAE > saline controls) were reversed. The agent was injected 30 min prior to the training on day 1 and PAE animals treated with ABT-239 had performances similar to the saline-treated control animals (Savage et al., 2010). The use of ABT-239 prior to *in vivo* electrophysiology recordings demonstrated that the agent improved DG LTP in PAE animals to levels similar to those of saline-treated control animals (Varaschin et al., 2010).

The researchers speculated that ABT-239 facilitated glutamate release. However, they added that there is also a possibility that the inhibition of H_3_R on cholinergic nerve terminals facilitates acetylcholine release which could facilitate glutamate release. Also, the ABT-239 agent could be acting on the inhibition of H_3_ autoreceptors that promote histamine release and could facilitate the excitation of glutamatergic neurons via H_1_ and H_2_ receptors (Varaschin et al., 2010). None of those studies found any effect of the agent on control animals.

A recent study using immunohistochemistry, biochemical, and radiohistochemical approaches investigated histamine H_2_R density and H_2_ receptor-effector coupling in several nerve terminal regions of moderate PAE rats. The results found no significant PAE-related differences in the density of H_2_R binding (Davies et al., 2019) in contrast to alterations in the H_3_Rs (Varaschin et al., 2018).

Together these data provide evidence for presynaptic alteration following PAE, specifically at moderate levels – which reinforces the fact that there is no known safe amount of alcohol to be taken during pregnancy. The identified presynaptic alterations in the context of moderate PAE highlight the significance of glutamate modulation, possibly through histamine receptor interactions. At this level of PAE investigations, there seems not to report on sex differences investigations.

Although there are not enough studies investigating the effects of procognitive agents on DG LTP in PAE, this discovery not only emphasizes the central role of glutamate in synaptic plasticity but also opens the door to the development of novel pharmacological interventions aimed at ameliorating the effects of PAE on cognitive function, which could have broader implications for individuals affected by FASD. Other research groups have investigated different pharmacological agents to enhance PAE-related cognitive deficits. Slice physiology studies have demonstrated reversal of cognitive deficits via positive modulation the AMPAR following administration of Aniracetam (allosteric modulator of AMPAR and Piracetam analogon) (Vaglenova et al., 2008; Wijayawardhane et al., 2008). Administration of Piracetam have demonstrated alleviation of PAE-related deficits in CA1-LTP, showed improvement in hippocampal cell viability and reduction of PAE-induced cell apoptosis (Yang et al., 2017).

## Summary, conclusion, and future considerations

This review highlights the extent to which a wide range of research has contributed to understanding some mechanisms and impairments associated with prenatal alcohol exposure. Various studies have consistently demonstrated alterations in synaptic plasticity following moderate levels of prenatal alcohol exposure, highlighting the need for ongoing investigations. Our understanding of the underlying neurobiological mechanisms and their impact on learning and memory processes has significantly improved. Animal models have demonstrated altered hippocampal neurogenesis following PAE (Berman and Hannigan, 2000; Mattson et al., 2019). Human studies on trajectories of brain development following PAE, have extensively investigated neuroanatomical differences in PAE and typically developing individuals. However, few studies have assessed brain development over time, which leaves a gap in understanding potential alterations in patterns of development following PAE (for review in humans and neuroimaging techniques, see Moore and Xia, 2022). Taken together, this information is important to provide evidence of the relationship between time of exposure, neural development, and behavior outcomes (Lange et al., 2019). However, many questions remain, particularly regarding sex differences, and more research is needed to determine how the specific timing and dose of exposure affect the mechanisms supporting LTP.

While it is well-accepted that the hippocampal formation is sensitive to any amount of developmental alcohol exposure, there is still a gap in understanding those deficits and the related behavioral and cognitive manifestations. Over the past few decades, technological advancement, better assessment tests, and multi-level investigations have provided great knowledge on the detrimental effects of PAE. Studies on LTP provide a well-established model system for evaluating prospective treatments and identifying potential neural bases of learning and memory deficits observed in PAE. Unfortunately, there have been relatively few studies in LTP conducted for a small number of laboratories in the past few decades and the mechanisms remain to be understood. The development of treatments clinically depends on more research in the area, which is needed to amplify our basic understanding of how PAE affects synaptic plasticity.

Promisingly, this review has shed light on potential interventions, encompassing both pharmacological and non-pharmacological approaches, offering potential information in mitigating the enduring impacts of PAE on cognitive functions.

However, it is imperative to emphasize that this review stresses the need for further research, in-depth investigations, and a better comprehension of how prenatal alcohol exposure negatively impacts synaptic plasticity and cognitive functions. The ultimate aim persists in advancing our understanding of the intricate consequences of PAE on the developing brain and cognitive processes, while also paving the way for the development of effective therapeutic interventions that can enhance the lives of those burdened by prenatal alcohol exposure.

## Author contributions

MG-G: Conceptualization, Writing—original draft. DH: Supervision, Writing—review and editing.
